# An analysis of performance bottlenecks in MRI preprocessing

**DOI:** 10.1093/gigascience/giae098

**Published:** 2025-03-03

**Authors:** Mathieu Dugré, Yohan Chatelain, Tristan Glatard

**Affiliations:** Concordia University, Department of Computer Science and Software Engineering, 1455 Blvd. De Maisonneuve Ouest, Montreal, Quebec H3G 1M8, Canada; Concordia University, Department of Computer Science and Software Engineering, 1455 Blvd. De Maisonneuve Ouest, Montreal, Quebec H3G 1M8, Canada; Concordia University, Department of Computer Science and Software Engineering, 1455 Blvd. De Maisonneuve Ouest, Montreal, Quebec H3G 1M8, Canada; Centre for Addiction and Mental Health, Krembil Centre for Neuroinformatics, 60 Leonard Ave, Toronto, Ontario M5T 0S8, Canada

**Keywords:** performance, profiling, MRI, preprocessing, neuroimaging

## Abstract

Magnetic resonance imaging (MRI) preprocessing is a critical step for neuroimaging analysis. However, the computational cost of MRI preprocessing pipelines is a major bottleneck for large cohort studies and some clinical applications. While high-performance computing and, more recently, deep learning have been adopted to accelerate the computations, these techniques require costly hardware and are not accessible to all researchers. Therefore, it is important to understand the performance bottlenecks of MRI preprocessing pipelines to improve their performance. Using the Intel VTune profiler, we characterized the bottlenecks of several commonly used MRI preprocessing pipelines from the Advanced Normalization Tools (ANTs), FMRIB Software Library, and FreeSurfer toolboxes. We found few functions contributed to most of the CPU time and that linear interpolation was the largest contributor. Data access was also a substantial bottleneck. We identified a bug in the Insight Segmentation and Registration Toolkit library that impacts the performance of the ANTs pipeline in single precision and a potential issue with the OpenMP scaling in FreeSurfer recon-all. Our results provide a reference for future efforts to optimize MRI preprocessing pipelines.

## Introduction

Preprocessing of magnetic resonance imaging (MRI) data is a critical step in neuroimaging. Although established preprocessing pipelines exist, they commonly require extensive amounts of computation and produce large volumes of output and intermediate data. Such requirements create challenges when studying large neuroimaging cohorts, and they limit clinical applicability when timely data analysis is needed. Therefore, the neuroimaging community is constantly seeking ways to improve the computational performance of MRI preprocessing pipelines. In this article, we characterize the computational cost of several commonly adopted MRI preprocessing pipelines, providing a reference benchmark for future efforts to optimize MRI preprocessing pipelines.

MRI is a standard tool used by neuroscientists to perform clinical diagnosis and for researchers to develop a better understanding of the brain. Three main MRI modalities exist: structural MRI (sMRI), functional MRI (fMRI), and diffusion MRI (dMRI). While other modalities such as electroencephalography (EEG), computed tomography (CT), and positron emission tomography (PET) exist, we focus on MRI for its broad adoption in research and noninvasiveness.

Neuroscientists developed various toolboxes to tackle the challenging task of preprocessing MRI data. We focus on the commonly accepted fMRIPrep [1] pipeline, as it is a comprehensive preprocessing application for both sMRI and fMRI. fMRIPrep combines several pipelines to produce a complete preprocessing pipeline. We study fMRIPrep’s subpipelines separately to provide a finer grained analysis of the performance bottlenecks.

To profile the pipelines, we use the Intel VTune tool [2], a multilanguage profiler with low performance overhead that provides runtime information at the level of functions. We profile the pipeline in single-threaded mode and using 32 threads to account for parallel executions. We aggregate subjects’ results for each pipeline to get an average performance profile.

## Materials and Methods

### Pipelines

fMRIPrep is a commonly used fMRI and sMRI preprocessing pipeline for neuroimaging, built on top of several well-known neuroimaging toolboxes such as Advanced Normalization Tools (ANTs) [3], FMRIB Software Library (FSL) [4], FreeSurfer [5], and Analysis of Functional NeuroImages (AFNI) [6]. Although processing steps may vary based on the type of input data, the sMRI pipeline uses ANTs BrainExtraction [7, 8] to perform intensity correction and skull-stripping of a T1-weighted image, FSL FAST [9] to segment the tissues of the extracted brain, and ANTs registrationSyN [8] to register the segmented brain. When enabled, FreeSurfer recon-all [10] is used for surface reconstruction. We benchmarked a recon-all command similar to fMRIPrep’s, which (i) performs autorecon1 without skull-stripping, (ii) imports an external skull-strip from ANTs brainExtraction, and (iii) resumes reconstruction with autorecon2 and autorecon3. The fMRI pipeline performs motion correction with FSL MCFLIRT [11], slice timing correction with AFNI 3dtshift [6], and coregistration with FSL FLIRT [11–13].

We profiled the entire fMRIPrep pipeline to get a coarse view of the pipeline’s performance bottleneck. To characterize the performance at a finer grain, we profiled the default subpipelines of fMRIPrep listed above. While we used the same subpipeline and order as in fMRIPrep, we used the default parameters for most of the subpipelines, which may vary from the default parameters of fMRIPrep. We omitted some subpipelines of fMRIPrep, as they were not compatible with our dataset. For example, slice-timing correction was already performed in our data.

ANTs brainExtraction and registrationSyN are available in single or double precision, leveraging the Insight Segmentation and Registration Toolkit (ITK) [14]. We profiled both versions to understand the performance benefits of using reduced precision in the pipelines.

### Data

To ensure that our performance profiles are inclusive of different populations, we used data with a wide range of age and equal distribution of sex. We used the OpenNeuro ds004513 v1.0.2 dataset [15]. The anatomical and functional data from 20 healthy individuals were acquired from 2 different cohorts. The within-subject cohort has 9 participants (mean age = 43 years, SD = 7 years; 4 females) with 2 sessions: eyes open and eyes closed. The replication cohort has 11 participants (mean age = 27 years, SD = 5 years; 6 females) with only the eyes open session. Each subject has an anatomical image with a resolution of 1 mm and a dimension of $160 \times 240 \times 256$ voxels (approx. 12 MiB). The subjects have 300 functional images taken at a 2-second interval with a resolution of 3 mm and a dimension of $64 \times 64 \times 35$ voxels (approx. 50 MiB).

### Profiling

Profiling MRI preprocessing pipelines raises a few challenges. Neuroimaging pipelines use several programming languages, including C, C++, Fortran, Matlab, and Python, with several pipelines using a combination of multiple languages. Pipelines are generally complex and computationally expensive.

The Intel VTune profiler addresses these challenges by offering multilanguage support, low performance overhead, and information on functions at the runtime level. However, common profiling challenges remain. Profilers require source code to be compiled with debug symbols to report interpretable information about the different functions and modules. Different input data can substantially impact the performance results because of conditional branching in the pipeline and convergence thresholds. We discuss our approach to these challenges in the next section.

First, to get human-readable information on function and module names in VTune, we recompiled each pipeline with debug information in a Docker image. For use on HPC systems, we created Apptainer [16](https://github.com/singularityhub/docker2singularity) images using Docker2Singularity. Listing 1 demonstrates the profiling of a pipeline by mounting the VTune binary to the Apptainer image during execution.

**Listing 1. Profiling a pipeline within an Apptainer container, using VTune profiler**.

#!/bin/sh singularity exec –cleanenv \

-B /intel/oneapi/vtune/latest/:/vtune \

-B ${SLURM_TMPDIR}:/data \

-B ${PROJECT_DIR}:${PROJECT_DIR} \

${SIF_IMG} \

/vtune/bin64/vtune \

-collect hpc-performance \

-knob enable-stack-collection=true \

-knob analyze-openmp=true \

-result-dir ${PROFILING_DIR} \

<script.sh>

We profiled the pipeline using 2 threading configurations: single-threaded and 32 threads. The single-threaded approach simplified the analysis and limited the potential I/O overhead on the profiling. The 32-thread approach used all available CPU cores from a computing node of our infrastructure to study the multithreading performance of the pipeline. This configuration provided a more realistic usage of the pipeline.

Before profiling each pipeline, we transferred the input data from the shared file system to the computing node. After profiling, we transferred back the output to the shared file system. This limited profiling variability is due to I/O contingency.

The data generated by the VTune profiler were written to a shared file system, to simplify postprocessing. In principle, this could have increased the profiling overhead. However, there was no impact, since the data generated by the VTune profiler were tiny and not written on the same file system as data produced by the pipelines.

### Metrics definition

VTune provides several metrics to characterize the performance of a pipeline. makespan measures elapsed time from the start to the end of the pipeline. The CPU time refers to the total time spent by the CPU, across all cores, to execute the pipeline.

The memory-bound metrics, in particular, focus on identifying bottlenecks where the CPU cannot proceed due to data unavailability. Specifically, memory-bound metrics count the number of cycles where the CPU is stalled waiting for in-flight memory demand loads to be satisfied. This indicates how efficiently or inefficiently the pipeline utilizes memory resources, highlighting scenarios where memory latency hinders performance.

L1 bound occurs when a demand load stalls CPU cores, even though the required data are already in the L1 cache. Similarly, L2, L3, and DRAM bound conditions occur when a demand load causes the CPU to stall due to a cache miss at a particular cache level, with the data instead residing in L2, L3, or DRAM, respectively. In each of these cases, the CPU is delayed while waiting for the requested data to be fetched from the next level in the memory hierarchy.

A store-bound condition arises when a store operation halts CPU execution, often due to insufficient memory bandwidth or resource conflicts. These metrics help isolate whether the performance bottleneck is due to memory bandwidth limitations or inefficient caching. Supplementary Fig. S1 shows the monitoring events and equation to derive the metrics defining the memory bound characterization [17] (itkVectorInterpolateImageFunction.h). We use the convention from [18](https://github.com/InsightSoftwareConsortium/ITK/issues/4593) for the equations.

The difference between the total memory-bound value and the sum of the other metrics indicates the proportion of CPU idle time caused by waiting for data that are missing from all cache levels, meaning the data are being retrieved from secondary storage, such as a disk.

### Infrastructure

We used the *Slashbin cluster* at Concordia University. The compute nodes are configured with two 16-core Intel Xeon Gold 6130 CPU @ 2.10 GHz, 250 GiB of RAM, 126 GiB of tmpfs, six 447 GiB SSDs with the XFS file system (no RAID enabled), Rocky Linux 8.9, and Linux kernel *4.18.0-477.10.1.el8_lustre.x86_64*.

## Results and Discussion

In this section, we present the aggregated profiling data collected using VTune. First, we present a performance overview of the pipelines, then analyze specific hotspots in the pipelines. Unless specified explicitly, we only present the profile data from using 1-thread since the results from 32-threads are similar. We aggregate the profiling results of each pipeline across all subjects to get an average performance profile. Consistently with [18], we hypothesized that the Pareto principle applied to performance of MRI preprocessing pipelines. We therefore only studied the functions with the largest contribution to CPU time, up to 80% cumulative total runtime of the pipeline. This heuristic allowed us to focus our efforts on the performance critical sections of a pipeline. We report the mean and standard deviation of the execution time for those functions.

### CPU time distribution across functions was long-tailed

**Figure 1:**
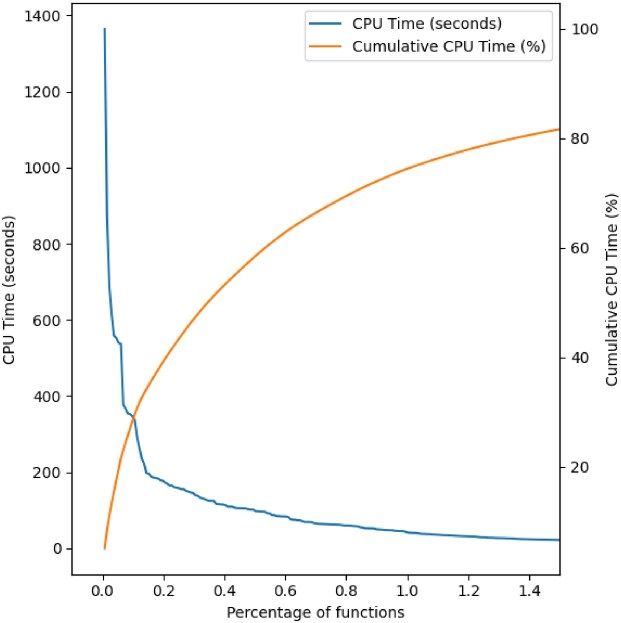
Distribution of CPU time for the functions. The left y-axis shows the average total CPU time spent in a function, while the right y-axis shows the cumulative CPU time percentage. The x-axis is the percentage of functions ordered by decreasing CPU time. Data include functions from all pipelines.

Figure 1 shows that 80% of the total CPU time was spent in less than 1.5% of the function across all pipelines. While consistent with the Pareto principle, the distribution of the functions’ CPU time did not follow a Pareto distribution. Using the *powerlaw* Python package [19] (https://github.com/freesurfer/freesurfer/issues/1199), we found the data fit a Zipf law with an exponent $\alpha =1.46$ (Supplementary Fig. S2). Therefore, the CPU time of a function was approximately inversely proportional to its rank. Thus, future efforts to optimize this minimal set of functions could bring significant performance improvements.

### Main bottleneck: Linear interpolation

Table 1 shows that interpolation was a critical part of all pipelines except FreeSurfer recon-all, contributing between 32.20% and 62.70% of the total CPU time. Overall, the number of functions using interpolation was low, with fewer than 20 in each pipeline. Few interpolation functions dominated CPU time. While each interpolation had low computational cost, the large amount of operations resulted in a significant bottleneck. Therefore, optimizing interpolation would bring substantial benefits to these pipelines.

**Table 1:** Contribution of interpolation to the pipelines’ total CPU time. The percentage is the averaged sum of the CPU time of functions using interpolation. The data include all functions from all pipelines.

Pipeline	# of functions with interpolation	% CPU time spent in interpolation
ants/brainExtraction	17	38.52
ants/brainExtraction-fp	17	38.37
ants/registrationSyN	7	43.67
ants/registrationSyN-fp	8	47.14
fsl/fast	1	42.97
fsl/mcflirt	3	32.90
fsl/flirt	4	62.07
freesurfer/reconall	10	3.71

### Impact of memory bounded functions

Table 2 shows that fetching data from disks was the main cause for starving the CPU, contributing an average of 6.60% across pipelines. Next, the L1 bound had the highest cache level contribution (5.58% average) to the memory bound of the pipelines. The ANTs pipelines had the highest L1 bound, followed by FreeSurfer recon-all and FSL MCFLIRT. L2 and L3 bound were low (0.88% and 1.17% average) for all pipelines, although higher for FreeSurfer recon-all. The DRAM bound was high for both FSL FLIRT (15.73%) and FreeSurfer recon-all (13.83%), followed by ANTs brainExtraction in double (5.08%) and single (2.48%) precision. Store bound was low for all pipelines. We note an interesting relationship between the low L1 bound and the high DRAM bound of FreeSurfer recon-all and FSL FLIRT compared to the other pipelines.

**Table 2:** Impact of data load on stalled CPU cycles. For each metric (Total, L1, L2, L3, DRAM, Store, and Disk Fetch), reported values are summations weighted by function CPU time, averaged over $n=20$ subjects, and collected in single-threaded mode. They represent the percentage of the total CPU time stalled for the metric.

Pipeline	Total memory bound %	L1 %	L2 %	L3 %	DRAM %	Store %	Disk Fetch %
ants/brainExtraction	22.61	7.08	0.47	0.81	5.08	0.23	8.94
ants/brainExtraction-fp	20.59	9.11	0.24	0.39	2.48	0.12	8.24
ants/registrationSyN	16.01	6.61	0.79	0.33	0.70	0.44	7.14
ants/registrationSyN-fp	17.97	9.65	0.30	0.08	0.30	0.16	7.48
fsl/fast	9.87	2.87	0.40	0.51	1.61	1.67	2.81
fsl/mcflirt	6.79	3.57	1.96	0.19	0.16	0.10	0.81
fsl/flirt	30.36	1.61	0.42	2.37	15.73	0.59	9.64
freesurfer/reconall	34.07	4.11	2.50	4.69	13.83	1.23	7.72
Average across pipelines	19.78	5.58	0.88	1.17	4.99	0.57	6.60

Optimizing the data access of the pipeline could reduce the number of data load. This could have a large impact on pipeline performance. Alternatively, the use of reduced precision could reduce the memory footprint and thus the memory bound. To reduce the memory bound impact of fetching data from disks, it could be possible to convert and store the data to a lower precision before preprocessing. In both cases, the impact of reduced precision on the accuracy of the pipelines should be studied.

### ANTs: Single vs. double precision

**Figure 2:**
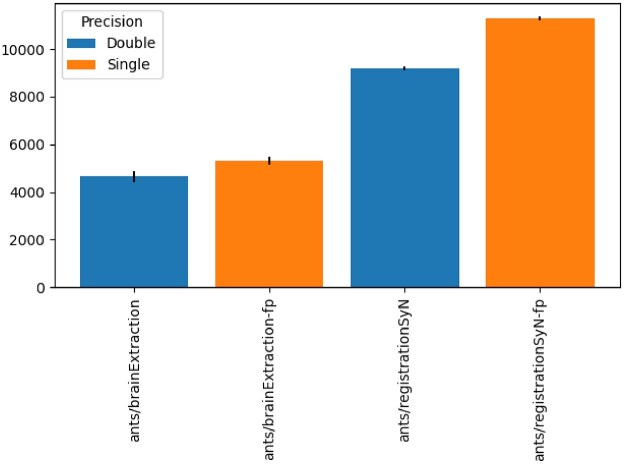
Comparison of makespan between double (blue) and single (orange) precision for ANTs brainExtraction and ANTs registrationSyN.

Figure 2 shows the makespan of ANTs brainExtraction and ANTs registrationSyN with double and single precision. For both pipelines, the makespan was significantly higher in single precision than in double precision. This was unexpected, as the floating-point arithmetic operations are supposed to be faster in single precision than in double precision [20](https://surfer.nmr.mgh.harvard.edu/fswiki/SystemRequirements).

**Figure 3:**
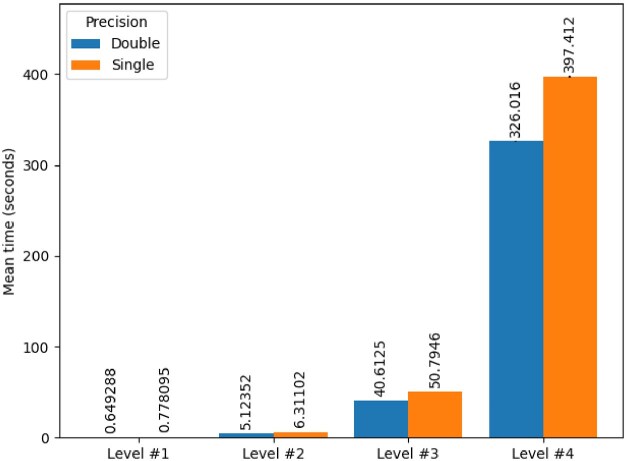
Average time per iteration for ANTs registrationSyN in double and single precision. Only the SyN Registration stage is shown as the 2 earlier stages were near zero time. The error bars show the standard deviation across $n=20$ subjects.

ANTs registrationSyN is a 3-stage process (Euler transform, affine transform, and SyN registration), where each stage is successively performed at 4 resolution levels with a specified number of maximum iterations. We focused on the SyN registration stage since it was substantially longer to execute than the other stages. The number of iterations between both versions of ANTs registrationSyN was similar. In fact, for the last 2 levels, the maximum specified number of iterations was reached across all but 1 execution. However, the single-precision version of ANTs registrationSyN took approximately 20% to 25% longer per iteration than the double-precision version for the last registration stage (Fig. 3). Therefore, the slowdown was not due to a slower convergence of the algorithm but rather to an increased execution time for each iteration.

After further analysis of the ANTs registrationSyN single-precision pipeline, we found that the use of templates in ITK’s C++ code forces the output of the interpolation function to be in double precision, with *EvaluateAtContinuousIndex* contributing most to the CPU time. As a result, the pipeline wastes CPU cycles by converting the input data to double precision in ITK and potentially casting it back to single precision in ANTs. We think this bug causes the slowdown. We tried recompiling a version of ITK to fix this issue, but we were unsuccessful because of the size and complexity of the code base. This example shows that the use of reduced precision is not trivial and requires a deep understanding of the code base. Benchmarks should be performed to ensure that the reduced precision does not impact the performance or accuracy of the pipeline. We reported this issue in the ITK GitHub repository.

### FreeSurfer: Thread synchronization

**Figure 4:**
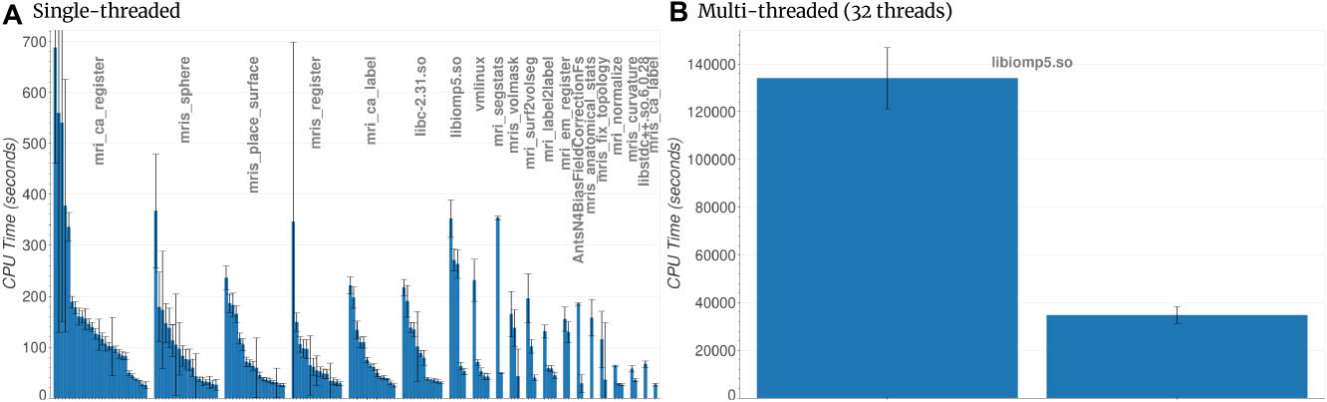
FreeSurfer recon-all analysis. Error bars show the standard deviation across *n* = 20 subjects. The x-axis shows the function ordered by decreasing CPU time grouped by module. We display only module names and omitted function names for clarity.

Figure 4 shows a significant difference in the pipeline profile between single-threaded and multithreaded executions. In the multithreaded executions (Fig. 4b), the OpenMP multithreading library was a major bottleneck for the pipeline, accounting for at least 76% of the pipeline runtime. The y-axis scale is multifolds larger than for the single-threaded execution, showing that the multithreaded execution was significantly slower than the single-threaded execution.

**Figure 5:**
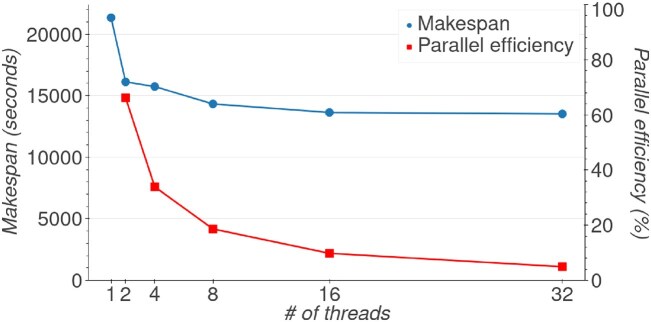
Makespan and parallel efficiency of FreeSurfer recon-all while varying the number of threads from 1 to 32. The left y-axis shows the makespan in seconds, while the right y-axis shows the parallel efficiency in percent. The log-scaled x-axis shows the number of threads.

While the makespan for FreeSurfer recon-all decreased with an increase in the number of threads, the benefits were limited (Fig. 5). The parallel efficiency decreased from 66% with 2 threads down to 5% with 32 threads. While Amdhal’s law limits the maximum parallel efficiency of the pipeline, Fig. 4B shows that most of the CPU time is spent in libiomp, which suggests that thread synchronization had a larger impact on parallel efficiency.

OpenMP offers multiple scheduling policies to assign chunks to threads, including *static* scheduling that assigns chunks to threads in a round-robin fashion, and *dynamic*, which assign chunks to threads as they become available for work. In the codebase, 83 out of 92 tasks use the *static* scheduling policy. This could lead to a load imbalance between the threads and thus a decrease in parallel efficiency. We speculate that *dynamic* scheduling could improve the parallel efficiency by assigning chunks based on the current load of the threads. Unfortunately, we failed to recompile FreeSurfer, having changed the *static* OpenMP scheduling to *dynamic*. This remains challenging as the codebase is large and complex, and the change in scheduling policy could lead to unexpected bugs. We opened an issue on FreeSurfer GitHub repository to report this problem.

Future work should study the different configuration of OpenMP scheduling in FreeSurfer recon-all to improve the parallel efficiency. We believe that significant performance improvements could be achieved by optimizing the thread synchronization. This would lead to faster runtime time and higher CPU usage ration in HPC allocations.

### Experiencing using VTune

The VTune profiler provided a rich and extensive amount of data for our analysis, which would have been difficult to retrieve otherwise. Thanks to its low performance overhead, we could profile the pipeline with minimal impact on the runtime, allowing the use of more subjects to get a more accurate performance profile. The documentation provided by VTune is extensive and well documented, which helped us to understand the different metrics and events available.

VTune requires a finalization step to query the data and generate a report. We found this step to be both time-consuming and resource intensive. For example, finalizing the results from a single FreeSurfer recon-all execution can require over 2 TB of RAM, while the developers recommend using between 8 and 16 GB of RAM to run the pipeline. This significant difference in resource requirement creates a challenge to profile pipeline with long runtime, because of the potential difficulty to get access to computing nodes with enough RAM. One way to mitigate this challenge is to lower the sampling rate during the profiling. However, the sampling rate would have to be significantly reduced for the finalization stage to require a similar amount of RAM (16 GB or less), which would lead to a potential loss of information in the profiling results. Because of these limitations, we could not collect the results of the entire fMRIPrep pipeline, and we only reported results for the subpipelines.

## Conclusion

In this article, we profiled several MRI preprocessing pipelines used in the popular fMRIPrep tool: ANTs brainExtraction, ANTs registrationSyN, FSL FAST, FSL FLIRT, FSL MCFLIRT, and FreeSurfer recon-all. A few functions were found to contribute significantly to the CPU time, presenting opportunities to optimize these functions and potentially achieve significant speed-up. It was discovered that linear interpolation is the main time bottleneck, with most pipelines being affected by memory bound. We discovered a bug in ITK, which leads to the version of ANTs registration in single precision to use double precision instead. A potential bug in FreeSurfer recon-all was found, which limits the benefit of multithreading with OpenMP. Last, we discussed the challenge of profiling long-running pipelines with VTune because of computational resource requirements.

The dataset we chose contains a wide range of ages and an equal distribution of sex. However, it contains only healthy subjects. It would be interesting to study the impact of image quality and pathologies on performance. Our profiling with VTune did not account for the spatiality (e.g., for loop) or temporality (e.g., start vs. end of convergence) of the functions. This information could provide additional insight for optimization. Future work could extend the depth of the analysis by including this information.

Future optimization efforts could focus on reduced precision techniques, to reduce memory bound and accelerate interpolation computation. We note that the use of reduced precision is not trivial, as seen with the ANTs bug. Therefore, careful attention would be required to find a balance between performance and accuracy. We also suggest for future work to study the different configurations of OpenMP scheduling in FreeSurfer recon-all to improve parallel efficiency. This would lead to faster execution time and higher CPU usage in HPC allocations.

We hope that this work serves as a reference for future work to optimize MRI preprocessing pipelines.

## Abbreviations

AFNI: Analysis of Functional NeuroImages; ANTs: Advanced Normalization Tools; CT: computed tomography; dMRI: diffusion magnetic resonance imaging; EEG: electroencephalography; fMRI: functional magnetic resonance imaging; FSL: FMRIB Software Library; HPC: high-performance computing; ITK: Insight Segmentation and Registration Toolkit; MRI: magnetic resonance imaging; PET: positron emission tomography; sRMI: structural/anatomical magnetic resonance imaging.

## Additional Files

**Supplementary Fig. S1**. Intel performance monitoring events and derived memory metrics.

**Supplementary Fig. S2**. Distribution of the functions’ CPU time compared to a Zipf’s law distribution with $\alpha =1.46$. The y-axis shows the average CPU time for a function. The x-axis shows the percentage of functions ordered by decreasing CPU time. The data include all functions from all pipelines.

**Supplementary Table S1**. FreeSurfer recon-all (32 threads): top functions accounting for 80% of the pipeline makespan.

giae098_Supplemental_Files

## Data Availability

Name: The OpenNeuro ds004513 v1.0.2 dataset DOI: http://dx.doi.org/10.18112/openneuro.ds004513.v1.0.2 License: Creative Commons Zero v1.0 Universal Public Domain Dedication Citation: [15] The scripts used in this study are available on GitHub, along with Jupyter notebooks to generate the figures. Name: mri-bottleneck Link: https://github.com/mathdugre/mri-bottleneck License: GPL-3.0 license Operating system: Platform independent Programming language: Bash and Python Other requirements: Intel VTune profiler [2] and Python packages: Bokeh, Jupyter, matplotlib, NumPy, Pandas, scipy PID: swh:1:snp:c9d107ac9c52f6baa8dbf02675a84861dd50f407; origin=https://github.com/mathdugre/mri-bottleneck Citation: [21] Name: Accompanying VTune results for “An Analysis of Performance Bottlenecks in MRI Pre-Processing” Link: https://doi.org/10.5281/zenodo.10987491 License: Creative Commons Zero v1.0 Universal Public Domain Dedication Citation: [22] The container images for our profiling experiments are available on Docker Hub: https://hub.docker.com/r/mathdugre/cmake https://hub.docker.com/r/mathdugre/intel-compilers https://hub.docker.com/r/mathdugre/afni https://hub.docker.com/r/mathdugre/ants https://hub.docker.com/r/mathdugre/fsl https://hub.docker.com/r/mathdugre/freesurfer https://hub.docker.com/r/mathdugre/fmriprep
