# Supplementary material for: An analysis of performance bottlenecks in MRI preprocessing
Source: Gigascience. 2025 Mar 3;14:giae098. doi: 10.1093/gigascience/giae098 (PMC11899568; doi:10.1093/gigascience/giae098)
Supplement: giae098_Supplemental_Files [file giae098_supplemental_files.pdf]

Supplementary Materials

$$C = CPU\_CLK\_UNHALTED.THREAD$$
$$S = CYCLE\_ACTIVITY.STALLS\_LDM\_PENDING$$
$$O = CYCLE\_ACTIVITY.STALLS\_L1D\_PENDING$$
$$T = CYCLE\_ACTIVITY.STALLS\_L2\_PENDING$$
$$W = MEM\_L3\_WEIGHT$$
$$H = MEM\_LOAD\_UOPS\_RETIRED.LLC\_HIT$$
$$R = MEM\_LOAD\_UOPS\_MISC\_RETIRED.LLC\_MISS$$
$$M = \frac{W \times R}{H + R}$$
$$N = \frac{H}{H + W \times R}$$

$$\%\ Memory\ Bound = \frac{S}{C} \times 100 \quad (1)$$
$$\%\ \text{L1}\ Bound = \frac{S - O}{C} \times 100 \quad (2)$$
$$\%\ \text{L2}\ Bound = \frac{O - T}{C} \times 100 \quad (3)$$
$$\%\ \text{L3}\ Bound = \frac{T \times N}{C} \times 100 \quad (4)$$
$$\%\ DRAM\ Bound = \frac{T \times M}{C} \times 100 \quad (5)$$

Fig. S1. Intel performance monitoring events and derived memory metrics

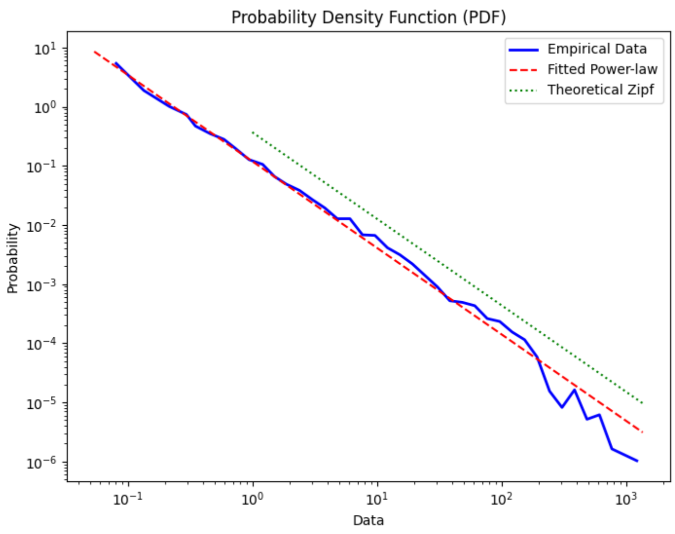

Fig. S2. Distribution of the functions' CPU time compare to a Zipf's law distribution with $\alpha = 1.46$. The y-axis shows the average CPU time for a function. The x-axis shows the percentage of functions ordered by decreasing CPU time. The data includes all functions from all pipelines.

	Module	Function	CPU Time (mean±std)
1	libiomp5.so	__kmp_fork_barrier	134064±12811
2	libiomp5.so	omp_set_lock	34816±3600

Table S1. FreeSurfer recon-all (32 threads): Top functions accouting for 80% of the pipeline makespan.
